# Regulation of Substrate-Target Distance on the Microstructural, Optical and Electrical Properties of CdTe Films by Magnetron Sputtering

**DOI:** 10.3390/ma11122496

**Published:** 2018-12-08

**Authors:** Peng Gu, Xinghua Zhu, Haihua Wu, Dingyu Yang

**Affiliations:** 1 College of Optoelectronic Technology, Chengdu University of Information Technology, Chengdu 610225, China; gupeng.cuit@foxmail.com (P.G.); xinghuazhu@cuit.edu.cn (X.Z.); zhouyanzhenzhen@foxmail.com (H.W.); 2 College of Intelligent Manufacturing, Sichuan University of Arts and Science, Dazhou 635002, China

**Keywords:** CdTe films, substrate-target distance, pinhole, optical properties, Hall-effect measurement system

## Abstract

Cadmium telluride (CdTe) films were deposited on glass substrates by direct current (DC) magnetron sputtering, and the effect of substrate-target distance (*D_ts_*) on properties of the CdTe films was investigated by observations of X-ray diffraction (XRD) patterns, atomic force microscopy (AFM), UV-VIS spectra, optical microscopy, and the Hall-effect measurement system. XRD analysis indicated that all samples exhibited a preferred orientation along the (111) plane, corresponding to the zinc blende structure, and films prepared using *D_ts_* of 4 cm demonstrated better crystallinity than the others. AFM studies revealed that surface morphologies of the CdTe films were strongly dependent on *D_ts_*, and revealed a large average grain size of 35.25 nm and a high root mean square (RMS) roughness value of 9.66 nm for films fabricated using *D_ts_* of 4 cm. UV-VIS spectra suggested the energy band gap (*Eg*) initially decreased from 1.5 to 1.45 eV, then increased to 1.68 eV as *D_ts_* increased from 3.5 to 5 cm. The Hall-effect measurement system revealed that CdTe films prepared with a *D_ts_* of 4 cm exhibited optimal electrical properties, and the resistivity, carrier mobility, and carrier concentration were determined to be 2.3 × 10^5^ Ω∙cm, 6.41 cm^2^∙V^−1^∙S^−1^, and 4.22 × 10^12^ cm^−3^, respectively.

## 1. Introduction

In recent years, silicon (Si) has been considered as the most important material for preparing film-form solar cells, due to its well-established preparation process and its theoretical conversion efficiency of Si-based solar cells being approximately 25% [1,2]. However, Si films exhibit low absorptivity in the range of 0.5–1.0 μm, and improving the absorptive capacity requires thicker films which would increase costs [3]. Furthermore, the energy band gap of Si films is only 1.12 eV, which cannot completely cover the radiation spectrum of sunlight [4]. Therefore, Si is not an optimal candidate for the fabrication of high-performance film-form solar cells. Due to their suitable energy band gap (1.45 eV) and high absorption coefficient (>10^5^ cm^−1^ in the visible range), CdTe films demonstrate distinctive merit among photovoltaic materials and achieve high photoelectric conversion performance, with a film thickness of only 1~2 μm [5,6,7,8]. However, some studies have proved that pinholes often appear on the surface of CdTe films and result in a leakage current, which increase the recombination rate of carriers in the pinhole defects, leading to a decrease in the filling factor (*FF*) and open-circuit voltage (*V_oc_*) [9,10]. In order to reduce the effect of pinholes, thick CdTe films were adopted as an absorption layer in solar cells. However, if the thickness of such films exceeds the diffusion length of the carriers, recombination of electron-hole pairs becomes significant and results in a decline in the performance of CdTe solar cells. Many techniques were implemented to prepare CdTe films, such as close-spaced sublimation (CSS) [11], molecular beam epitaxy (MBE) [12], pulsed laser deposition (PLD) [13], and magnetron sputtering technology [14]. Typically, CdTe films are deposited on substrates by CSS, due to the fast rate of growth within a few minutes and high material usage (up to 70%). However, this process requires a high temperature of 550–625 °C [15]. Magnetron sputtering technology is a good alternative candidate for fabricating CdTe films because of its high deposition rate, better adherence between the film and the substrate, and homogeneous coating for large areas, as well as the possibility of deposition at a low temperature [16,17,18].

In previous studies, sputtering conditions, including substrate temperature [19], discharge current [15], substrate bias [20], and other factors [21,22,23] were systematically investigated by many researchers, indicating that the crystallinity of CdTe films can be optimized by adjusting the sputtering parameters; however, the function of substrate-target distance on propertied of CdTe films was few reported. Shin et al. proved that substrate-target distance was a critical parameter affecting the growth of films, due to the kinetic energy of arrived atoms on the substrate [24]. Thus, research on the influence of substrate-target distance on properties of CdTe films should be further discussed. 

In this study, CdTe films were deposited on glass substrates by direct-current (DC) magnetron sputtering technology using different sputtering distances. The results suggested that the structural, morphological, optical, and electrical properties of CdTe films were strongly affected by the substrate-target distance (*D_ts_*), and films prepared using *D_ts_* of 4 cm demonstrate optimal performance in various aspects. This research provides a straightforward method for preparing CdTe films with high crystallinity, and provides a reference for preparing high-performance CdTe solar cells.

## 2. Materials and Methods

**Figure 1:**
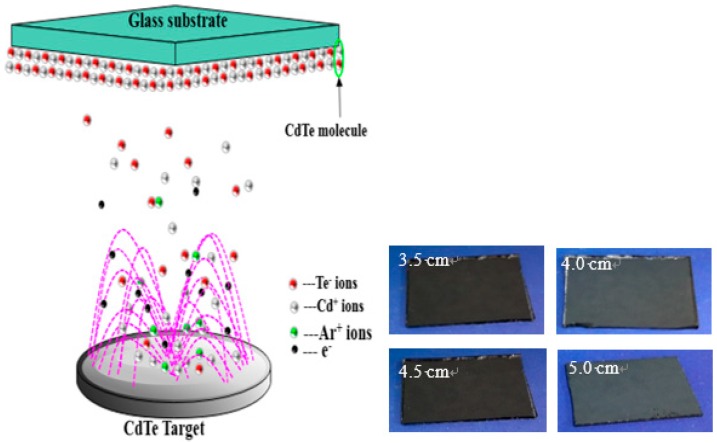
Schematic diagram of the sputtering process of cadmium telluride (CdTe) films.

Before the sputtering process, glass substrates were cleaned according to Reference [25]. CdTe (99.9% purity, Dumoers New Materials Inc., Beijing, China) was used as raw target material, and argon (99.99% purity, Niuruide Inc., Wuhan, China) was used as the sputtering gas. The argon was ionized by an electrostatic field, producing Ar^+^ ions and secondary electrons, after which the CdTe target was sputtered by the Ar^+^ ions at high kinetic energy, producing Cd^+^ and Te^−^ ions, which were deposited on the glass substrates and ultimately formed CdTe. Referring to Lu et al. [26], and Natarajan et al. [27], it has been found that if the sputtering distance exceeds 6 cm, the crystallinity of films deteriorates. Therefore, appropriate distances varying from 3.5 to 5 cm with intervals of 0.5 cm were implemented to deposit the CdTe films. A schematic diagram of the sputtering process is shown in Figure 1, and specific sputtering parameters are exhibited in Table 1. 

The crystal structure and crystallization were analyzed by X-ray diffraction (XRD) (SIEMENS D500 system, SIEMENS, Beijing, China) using Cu Ka radiation (λ = 0.1541 nm) and a scanning rate of 2°/min. The surface morphologies and roughness of the CdTe films were studied by atomic force microscopy (AFM) (Asylum Research Cypher, Oxford Instruments Technology Co., Ltd., Shanghai, China). Optical properties were investigated by the spectrophotometer (JASCO, V-670, Jiashike Trading Co., Ltd., Shanghai, China), and the electrical properties of films were measured using the Hall-effect measurement system (HMS-5000. Shanghai Zaide Semiconductor Technology Co., Ltd., Shanghai, China).

## 3. Results and Discussion

**Figure 2:**
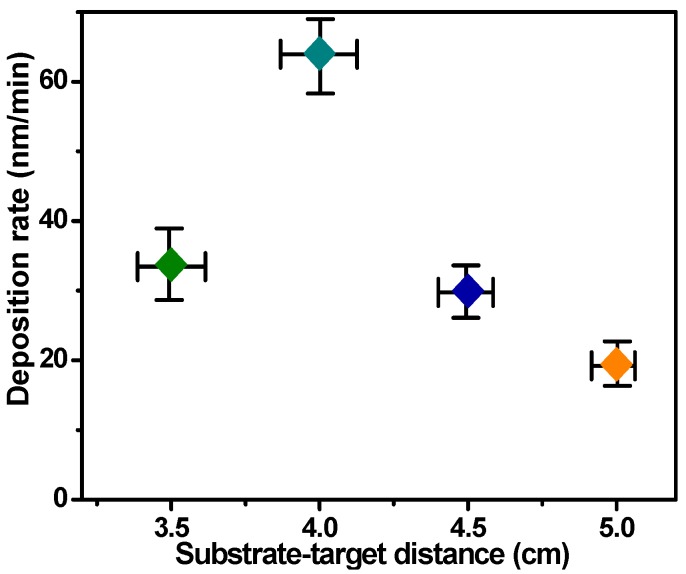
Deposition rate of CdTe films prepared by different sputtering distances.

Figure 2 displays the deposition rate of the CdTe films using different values of *D_ts_*. It can be clearly seen that the deposition rate of films was strongly affected by *D_ts_* and exhibits a maximum of 24.4 nm/min at *D_ts_* = 4 cm. There are a greater number of collisions and a large amount of scattering between the sputtering particles, such as Cd^+^, Te^−^, and Ar^+^ in the glow discharge area when *D_ts_* = 5 cm, resulting in detectable energy loss for Ar^+^ and CdTe molecules [28,29]. This effect reduces sputtering yield and decreases the kinetic energy of CdTe molecules, which inhibits the growth of CdTe films and leads to a low deposition rate. However, when *D_ts_* decreased, the adverse effect caused by the large *D_ts_* value lessened, and the deposition rate of the films improved. It is noted that when *D_ts_* decreased from 4 to 3.5 cm, the deposition rate of the films did not improve any further, due to the excessive kinetic energy of the CdTe molecules [30]. The CdTe film thicknesses were 2024, 3843, 1798, and 1167 nm, respectively, as *D_ts_* increased from 3.5 to 5 cm. 

**Figure 3:**
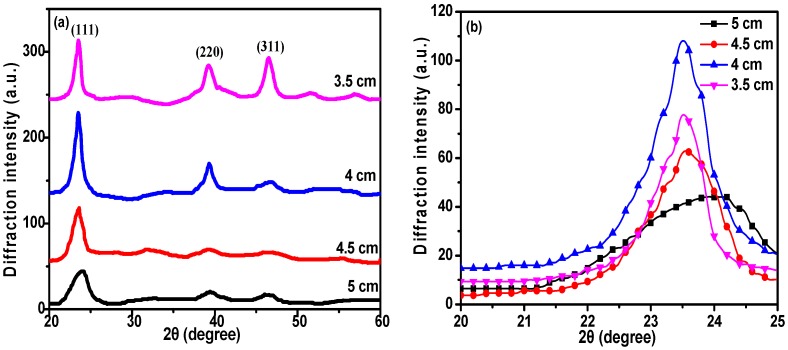
X-ray diffraction (XRD) analysis of CdTe films deposited at different substrate-target distances. (**a**) XRD patterns, and (**b**) comparison of the main peak for all samples.

The crystal structures of the CdTe films are shown in Figure 3a and exhibit three distinguishable peaks for each sample, representing planes (111), (220), and (311), respectively [31,32]. The peak corresponding to the (111) plane shows a significantly larger diffraction intensity compared to the other peaks, confirming that the CdTe films exhibit a preferred orientation along the (111) plane, which indicates the close packing direction of the zinc blende structure [33]. The observed preference for this orientation shows that it gradually strengthens when *D_ts_* decreases from 5 to 4 cm, but abruptly weakens at *D_ts_* = 3.5 cm, indicating that high crystallinity is achieved by the CdTe films at *D_ts_* = 4 cm. A shift of the (111) plane peak is observed in Figure 3b, which indicates a change in interplanar distance (d) of the (111) plane, caused by lattice distortion [34]. The average crystallite size (D) can be calculated by Scherrer’s equation [35]:

(1) $$D=\frac{0.9\lambda }{\beta cos\theta }$$

where *λ* is the X-ray wavelength (*λ* = 1.5406 Å), *β* is the full width at half maximum of the main peak in radians, and *θ* is the Bragg diffraction angle in degrees. The micro-strain (*ε*) and dislocation density (*σ*) were evaluated by the following equations [36]:

(2) $$\varepsilon =\frac{\beta cos\theta }{4}$$

(3) $$\sigma =\frac{1}{D^{2}}$$

**Figure 4:**
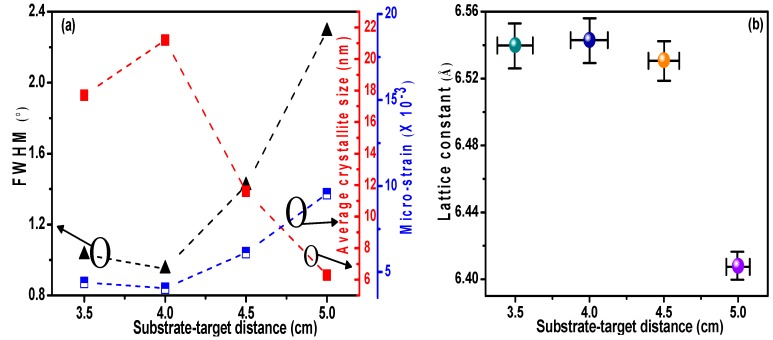
Crystal parameters of CdTe films prepared at various substrate-target distances. (**a**) Full width at half maximum, average crystallite size, and micro-strain; (**b**) lattice constant.

Changes in the crystal parameters of the CdTe films are shown in Figure 4. Films prepared at *D_ts_* = 4 cm exhibited a large crystallite size and low micro-strain, corresponding to 21.2 nm and 4.06 × 10^−3^, respectively. This indicates that the crystal structures of CdTe films are strongly dependent on *D_ts_*, attributing to the effect of the number of collisions and scattering among sputtering particles on the growth of the films. The suitable value of kinetic energy and weaker collisions of CdTe molecules were found to improve crystallinity. The films’ growth most likely developed compressive stress that pushed the films’ surface outwards, thereby making the curvature of the thin film/substrate system more convex and increasing the value of the lattice constant, which explains how the increase in lattice constant was observed with an improvement in the CdTe films’ crystallinity [6]. The specific crystal parameters of the CdTe films are shown in Table 2. 

**Figure 5:**
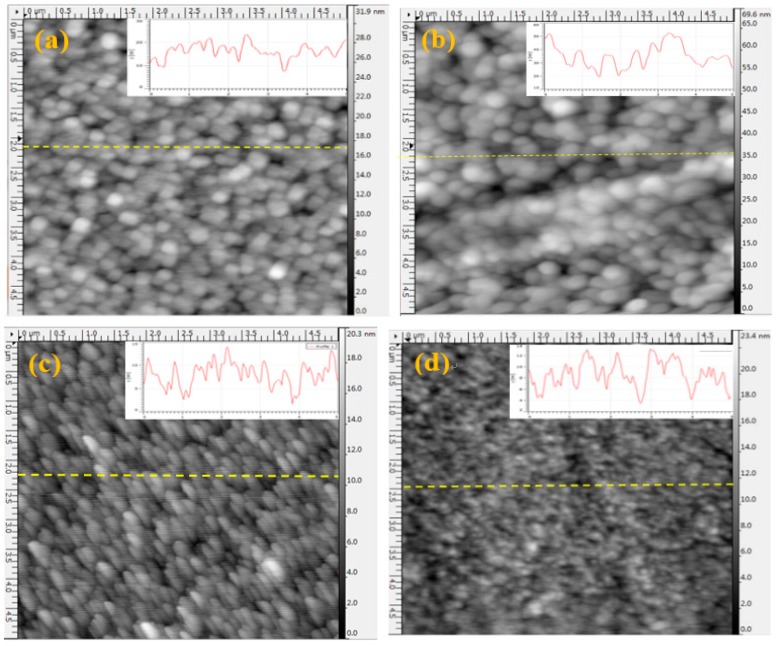
Two-dimensional surface morphologies of CdTe films prepared at different substrate-target distances, *D_ts_*. (**a**) 3.5 cm; (**b**) 4 cm; (**c**) 4.5 cm; and (**d**) 5 cm.

**Figure 6:**
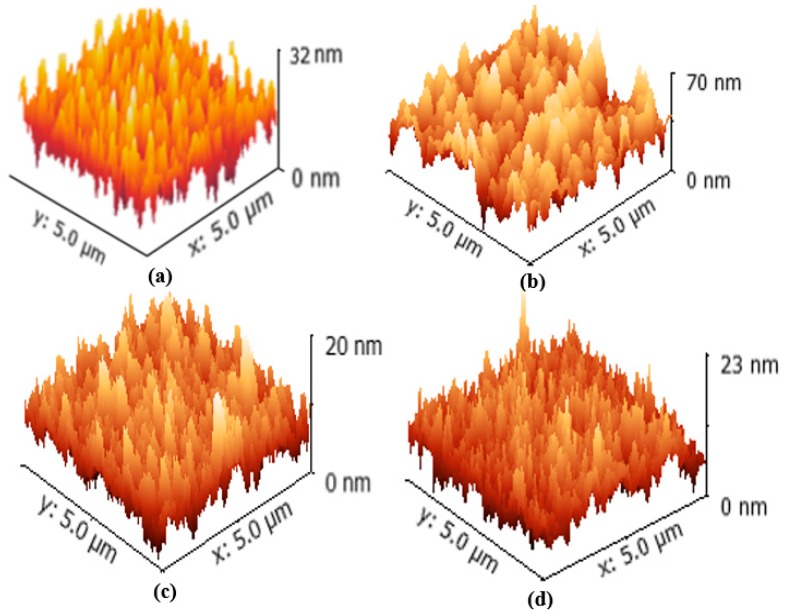
Three-dimensional surface morphologies of CdTe films deposited at different substrate-target distances, *D_ts_*. (**a**) 3.5 cm; (**b**) 4 cm; (**c**) 4.5 cm; and (**d**) 5 cm.

The two- and three-dimensional surface morphologies of the CdTe films are exhibited in Figure 5 and Figure 6. The average grain size and root mean square (RMS) roughness of the films were calculated by Gwyddion image software, revealing a smooth and flat CdTe film surface for *D_ts_* = 5 cm that yielded an average grain size of 7.88 nm and an RMS roughness of 2.29 nm. As *D_ts_* decreases from 5 to 4 cm, the outline of the grains becomes distinguishable and exhibits a spherical morphology. Additionally, compact films deposited at *D_ts_* = 4 cm (with an average grain size of 35.25 nm and RMS roughness of 9.66 nm) are obtained, which effectively prevent the occurrence of pinholes on the film surface. However, for *D_ts_* = 3.5 cm, the surface state of the CdTe films (with an average grain size of 16.26 nm and RMS roughness of 3.89 nm) was not a further improvement, due to the excessive kinetic energy of the CdTe molecules and significant bombardment from sputtering particles. The inset images in Figure 5 represent the profiles of film surfaces that demonstrate the fluctuation of morphology and surface roughness. Furthermore, the specific parameters are listed in Table 3.

**Figure 7:**
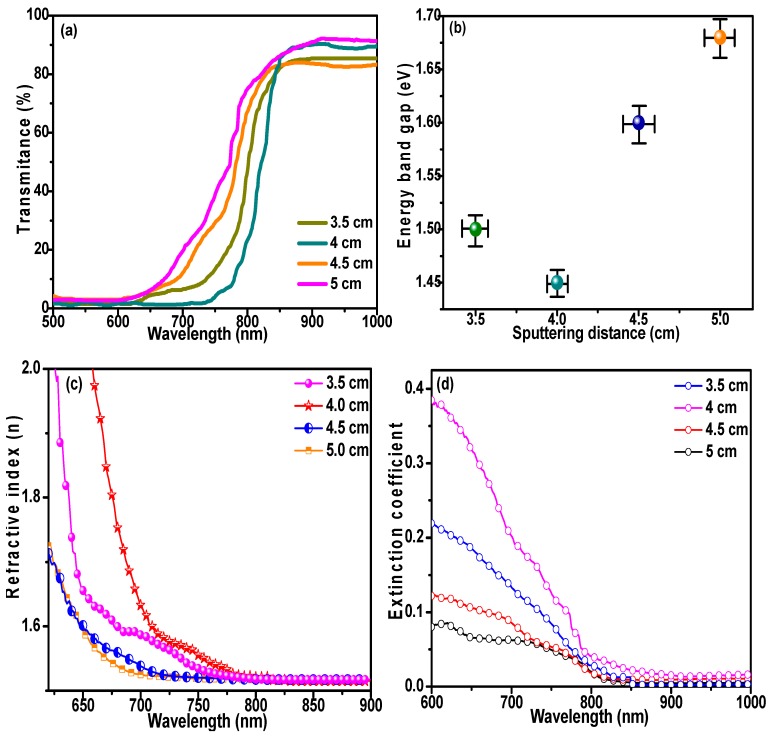
Optical properties of CdTe films prepared at different substrate-target distances. (**a**) Transmission spectrum; (**b**) energy band gap; (**c**) refractive index (n); and (**d**) extinction coefficient.

CdTe films are used as the absorbing layer in solar cells, and their optical properties directly affect device performance and play a critical role in photoelectric conversion efficiency [37]. CdTe, with its narrow energy band gap (*E_g_*) and high extinction coefficient, is an optimal candidate for the fabrication of high-efficiency solar cells. Transmission spectra of CdTe films are presented in Figure 7a, revealing no obvious change in transmittance for wavelengths (*λ*) shorter than 650 nm. However, if *λ* increases, the transmittance rises dramatically to greater than 80%, as *λ* exceeds 850 nm. This result is mainly determined by the relationship between the energy of the incident light and the band gap of CdTe films. The band gap of the samples was calculated by the following formula [25]:

(4) $${\left(\alpha hv\right)}^{2}=A\left(hv-E_{g}\right)$$

(5) $$\alpha =\frac{ln\left(\frac{1}{T}\right)}{d}$$

where *α* is the absorption coefficient, *E_g_* is the energy band gap, *hv* is the photon energy, *A* is a constant, *d* is the film thickness, and *T* represents the transmittance. The results are shown in Figure 7b. CdTe films prepared at a *D_ts_* of 4 cm exhibited the minimum value (*E_g_* = 1.45 eV) due to the quantum size effect [38,39]. It is believed that a decrease in *E_g_* can improve the optical properties and expand the spectral response range. Meanwhile, the refractive index (*n*) of the CdTe films was calculated based on the Swanepoel method [40]:

(6) $$n=\sqrt{H+\sqrt{H^{2}-S^{2}}}$$

(7) $$H=\frac{4S^{2}}{\left(S^{2}+1\right)T^{2}}+\frac{S^{2}+1}{2}$$

Here, *S* refers to the substrate refractive index (1.52 for glass), *T* is the transmittance, and *H* is the Swanepoel coefficient. It can be seen in Figure 7c that the refractive index gradually increases as *D_ts_* drops from 5 to 4 cm, which we attributed to the increase in grain size and decrease in porosity of the CdTe films. However, where *D_ts_* decreases from 4 to 3.5 cm, the refractive index of the films only dropped slightly due to the reduced crystallinity. Additionally, a decrease in *E_g_* can effectively increase the refractive index [6]. It is well-known that the extinction coefficient (K) provides information about the absorption of light in a medium due to inelastic scattering [32]. The extinction coefficient of the CdTe films was calculated by the following formula [41]:

(8) $$K=\frac{\alpha \lambda }{4\pi }$$

As shown in Figure 7d, changes in the extinction coefficient exhibited a similar trend to changes in the refractive index. CdTe films deposited with *D_ts_* of 4 cm featured a higher extinction coefficient, because highly crystalline films with low porosities can effectively enhance the interaction between the film and incident light. A lower *k* value was observed for films prepared at *D_ts_* = 5 cm, which indicates excellent surface homogeneity and smoothness of these deposited CdTe films [32].

**Figure 8:**
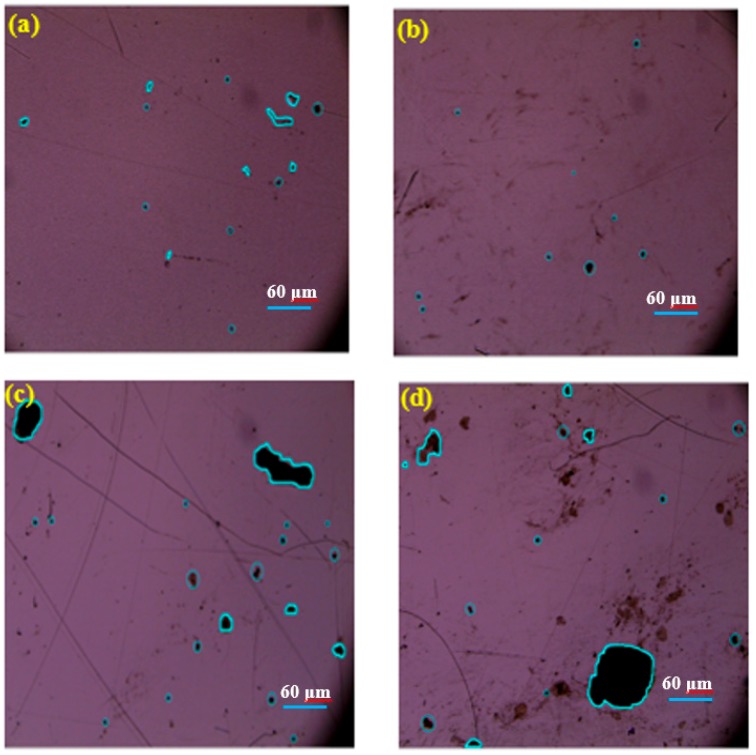
Optical images of CdTe films prepared at different sputtering distances. (**a**) 3.5 cm; (**b**) 4 cm; (**c**) 4.5 cm; and (**d**) 5 cm.

Research has proven that the formation of pinholes in CdTe films reduces the efficiencies of solar cells based on CdTe films due to shunting effects that produce short-circuits. Pinhole areas contain various defects that enhance the process of capturing electron-hole pairs, resulting in a lower effective number of photo-generated carriers [42,43,44]. Before investigating the influence of pinholes on the electrical properties of CdTe films, the mechanism of pinhole formation should first be explained. Pinholes can be divided into two categories: natural and artificial [45]. The former is mainly associated with the coalescence of grains, while the latter is attributed to damage from handling after deposition, resulting in scuffs and scratches on the film surface. The distribution of pinholes on the film surface are shown in Figure 8. It was found that the number of pinholes formed dropped significantly to the lowest density of pinholes (defined as the number of the pinholes area divided by the total area), which was 0.026 when *D_ts_* was 4 cm. It was concluded that the number of pinholes on the CdTe film surface could be significantly decreased by adjusting the sputtering distance, instead of requiring technology such as pinhole fillers with negative photoresist.

**Figure 9:**
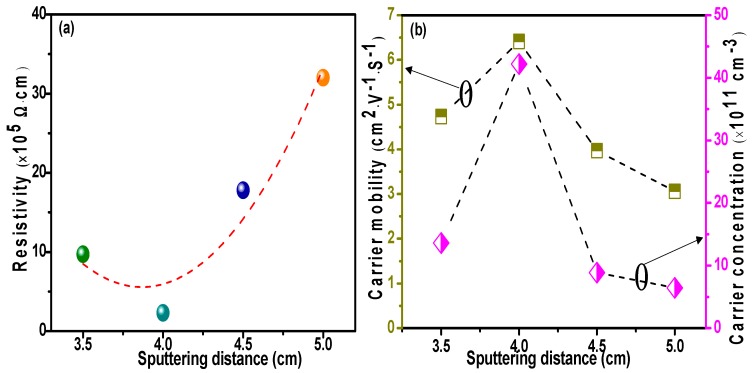
Electrical properties of CdTe films prepared at various substrate-target distances. (**a**) Resistivity; (**b**) carrier mobility.

The electrical properties, including resistivity, carrier mobility, and carrier concentration of CdTe films are key parameters for their implementation in solar cells, and were tested by a Hall-effect measurement system. Wang et al. reported that the resistivity, Hall mobility, and carrier concentration of films were mainly dependent on crystallinity and grain boundaries [46]. High crystallinity and a low number of grain boundaries in films typically improve electrical properties, because the grain boundaries act as a source of trap states, resulting in more charge carriers recombining in these regions, effectively increasing resistivity and also determining carrier mobility by structural defects due to carrier scattering and trapping [46,47]. The optimal resistivity, Hall mobility, and carrier concentration of the CdTe films prepared using a *D_ts_* of 4 cm were determined as 2.3 × 10^5^ Ω∙cm, 6.41 cm^2^∙V^−1^∙S^−1^, and 4.22 × 10^12^ cm^−3^, respectively, due to the full growth of CdTe films. The results are shown in Figure 9 and Table 4. Furthermore, the electrical properties of films optimized by adjusting the substrate-target distance are comparable to or better than others [48,49], indicating the effectiveness of this approach.

## 4. Conclusions

CdTe films were successfully deposited on glass substrates by direct-current magnetron sputtering technology, and the effect of sputtering distance on the structural, morphological, optical and electrical properties of CdTe films were investigated and discussed. The findings suggest that CdTe films prepared using a *D_ts_* of 4 cm exhibited the best crystallinity, attributed to a suitable kinetic energy value of CdTe molecules and weak collisions between the sputtering particles. CdTe films deposited at a *D_ts_* of 4 cm showed better optical properties in terms of refractive index and extinction coefficient, due to larger grain size and lower porosity. It was also found that modifying the substrate-target distance could effectively reduce pinhole formation on the film surface. Finally, the electrical properties of the CdTe films were tested using the Hall-effect measurement system, and films made with *D_ts_* = 4 cm that exhibited better electrical properties with the resistivity, carrier mobility, and carrier concentration of CdTe films, measured to be 2.3 × 10^5^ Ω∙cm, 6.41 cm^2^∙V^−1^∙S^−1^, and 4.22 × 10^12^ cm^−3^, respectively, due to high crystallinity and few grain boundaries. Overall, this work provides a meaningful reference for the preparation of CdTe solar cells demonstrating high performance of photoelectric conversion efficiency.

## Figures and Tables

**Table 1:** Deposition parameters of CdTe films prepared by magnetron sputtering.

Item	Parameter
Sputtering power	40 W
Deposition time	60 min
Substrate temperature	Room temperature
Sputtering gas	Ar: 50 sccm
Target	CdTe (diameter: 100 mm, thickness: 4 mm, 99.999%)
*D_ts_*	3.5, 4, 4.5, 5 cm

**Table 2:** Crystal parameters of CdTe films prepared at different substrate-target distances, *D_ts_*.

*D_ts_* (cm)	2*θ* (°)	*d* (Å)	FWHM ^1^ (°)	*D* (nm)	*E* × 10^−3^	*σ* × 10^15^ (lines/m^2^)
3.5	23.54	3.776	1.03	17.7	4.39	3.2
4	23.52	3.778	0.95	21.2	4.06	2.2
4.5	23.56	3.771	1.42	11.6	6.12	7.4
5	24.00	3.700	2.29	6.3	9.54	25.2

^1^ FWHM: full width at half maximum.

**Table 3:** Surface morphology parameters of CdTe films prepared at different substrate-target distances.

*D_ts_* (cm)	Average Grain Size (nm)	RMS Roughness (nm)
3.5	16.26	3.89
4	35.25	9.66
4.5	8.62	2.34
5	7.88	2.29

**Table 4:** Specific electrical parameters of CdTe films.

*D_ts_* (cm)	Resistivity (Ω∙cm)	Carrier Mobility (cm^2^∙V^−1^∙S^−1^)	Carrier Concentration (cm^−3^)
3.5	9.7 × 10^5^	4.73	1.36 × 10^12^
4	2.3 × 10^5^	6.41	4.22 × 10^12^
4.5	1.78 × 10^6^	3.97	8.87 × 10^11^
5	3.2 × 10^6^	3.06	6.43 × 10^11^
